# Implementing Case Management in New York State’s Partnerships for Publicly Funded Breast Cancer Screening

**Published:** 2008-03-15

**Authors:** Patricia P Lillquist

**Affiliations:** Bureau of Chronic Disease Epidemiology and Surveillance, New York State Department of Health

## Abstract

**Introduction:**

This research aimed to explore differences in the implementation of case management among local breast cancer screening partnerships in New York State after changes in federal public policy in 1998 and to achieve a better understanding of case management in a new and distinct practice setting. Capacity and willingness to implement change were theorized to explain local differences in implementation. Local breast cancer screening programs that received federal funding through the New York State Department of Health were invited to participate in the study.

**Methods:**

A mail survey was administered to the directors of New York's 53 local breast cancer screening partnerships in 2003. The survey included questions about willingness and capacity to implement case management and a scale to assess case management program philosophy. Factor analysis and correlations were used to compare willingness and capacity with differences in implementation.

**Results:**

Two common factors — task focus and self-identity focus — were identified as factors that differentiated case management programs. Task-focus partnerships undertook a broader range of tasks but were less likely to report autonomy in making program changes. Self-identity partnerships were less likely to report difficulties with other agencies and scored highly on innovation, involvement in work, and interest in client service. Having a nurse as the case manager, being aware of the standards of case management, and providing health education were associated with both task focus and self-identity focus.

**Conclusion:**

The study identified distinct styles of implementation. These styles have implications for the breadth of services provided, such as whether client-level services only are offered. Interagency coordination was facilitated in partnerships with comprehensive case management.

## Introduction

### Case management

Case management was developed to address fragmentation in service delivery and is rooted in the social casework tradition (1,2). The theoretical basis for case management is systems theory (3), as reflected by standards established by the Case Management Society of America (4) and the National Association of Social Workers (5), with case managers intervening at the individual, agency, community, and political or policy levels. Case management is used for clients who have extensive, complex, and ongoing needs, such as frail, elderly people or people with severe mental illness (6,7). In practice, case management usually takes place at the individual level and seldom occurs at the agency, community, or political or policy levels because of the immediate needs of clients and the requirements of the organization or supervisor (5).

Little consensus exists on what constitutes case management in traditional settings, such as mental health or aging services, let alone in new settings, such as cancer screening (8-11). Tasks performed by case managers are not always clearly delineated in services that offer case management (12). Holloway and Carson (2) note that literature on the effectiveness of case management in traditional settings is limited primarily to "anecdotal reports and poor quality research studies." Many of the models in the literature are linked so closely to specific client groups that model elements cannot be easily conceptualized for use in other settings. Choices in how to implement case management determine the structure and, therefore, the breadth and effectiveness of any case management program (13).

### Federal and state policy implementation

In preliminary unpublished work for this study, the author reviewed records from the Centers for Disease Control and Prevention's National Breast and Cervical Cancer Early Detection Program (NBCCEDP) and from the New York State Department of Health (NYSDOH) and spoke with staff members at both agencies to learn how case management policy for breast cancer screening programs was conceptualized at the federal and state levels and communicated to local programs. A summary of these findings follows.

States participating in the NBCCEDP were prohibited by federal legislation from using national funding for treatment but were required to ensure their clients received diagnostic testing and treatment as needed. In 1996, NBCCEDP sponsored a case study to determine how states identified resources and obtained these services for clients; this study found the process of identifying resources for follow-up care to be labor-intensive and only a short-term solution for providing client care, in part because approaches for delivering services were fragmented (14). In 1997, NBCCEDP surveyed states about their use of case management and presented a video conference on case management for states and providers. An internal NBCCEDP committee convened in September 1998 recommended that NBCCEDP pursue case management as a component of the NBCCEDP, review the literature on case management effectiveness, and draft guidelines for local programs. The subsequent literature review included only articles describing a systems-oriented approach (15). Committee meeting transcripts show that some NBCCEDP committee staff members viewed case management as instrumental in addressing psychosocial and socioeconomic needs that impede diagnosis and treatment of breast cancer and as distinct from clinically oriented disease or care management.

In October 1998, the Women's Health Research and Prevention Amendments of 1998 (Pub L No. 105–340) amended the law that established the NBCCEDP to add the words "support services such as case management" (16). With passage of this amendment, NBCCEDP could move forward in identifying key elements of case management policy for its participating states, including assessment, planning, coordination, monitoring, evaluation, and resource development.

An NBCCEDP memorandum to states in 1999 indicated that approximately $5.6 million in supplemental funding for case management was available nationwide. Ultimately, state breast cancer screening programs were asked to implement case management services for women with abnormal breast screening results without additional federal funding.

In New York, publicly funded breast cancer screening is organized locally by 53 partnerships comprising more than 2300 partner agencies that provide more than 40,000 mammograms to underserved women each year. When NBCCEDP began emphasizing case management in 1998, NYSDOH staff reported in interviews that they and staff in other states believed clients were well served by the tracking and follow-up services already in place — without case management — to ensure timeliness of testing, diagnosis, and treatment of breast cancer. Nonetheless, NYSDOH moved forward to require case management, viewing NBCCEDP's 1999 policy report, its conference calls about case management with states in 2000, and its 2000 operational plan for case management as a mandate.

In 2001, NYSDOH mailed the following definition of case management to partnerships: "activities that can increase client adherence to screening, diagnostic and treatment recommendations." NYSDOH described case management as having a goal similar to that of tracking and follow-up, the previous model for ensuring timeliness of diagnostic care, but involving "a more direct and personal level of support for clients at risk for not obtaining recommended diagnostic or treatment services." This definition addressed primarily client-level activities and did not include system-level activities such as resource development or advocacy. In interviews with key informants, NYSDOH staff indicated that partnerships were asked to focus on client activities so the partnerships would not be burdened with additional tasks without corresponding funding. Other NYSDOH documents distributed to partnerships focused on the time frames for completing case management activities, reflecting the idea that case management grows out of tracking women with abnormal test results. After the initial year of case management implementation among the partnerships, state funding for case management was provided in 2002.

In 2004, the author suggested that case management in publicly funded breast cancer screening programs differed from case management in traditional settings, such as in case management of chronically mentally ill individuals (17). Differences include a limited time in which to interact with clients; a lack of opportunity to function as a client advocate or influence the medical care system; and preconceived ideas about clients' problems, such as defining the client's problem as noncompliance (17). The research presented examines case management in local breast cancer screening partnerships within New York for a better understanding of case management in a new and distinct practice setting.

## Methods

### Theoretical framework

The communications model of intergovernmental policy implementation, a systems model, was used as a theoretical framework for this analysis (18). The original model was modified to separate implementation at the state level from implementation at the local level because breast cancer screening in New York is provided through local-level partnerships (Figure). Decisional outcomes are viewed as the desire or willingness to implement policy and as separate from the capacity to do so. This study focuses on the relationship between willingness (*local decisional outcome*) and capacity (*local capacity*) to implement case management and actual local implementation of case management.

**Figure 1:**
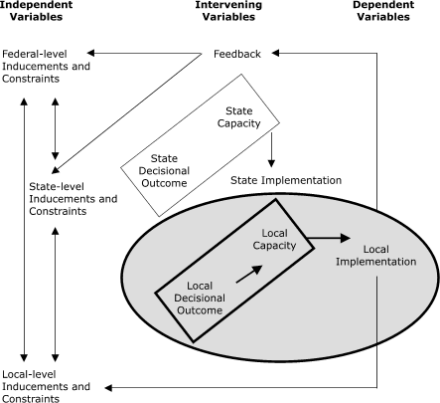
Conceptual model for study on case management among 53 partnerships providing breast cancer screening services in New York. The study focuses on the relationship between willingness (*local decisional outcome*) and capacity (*local capacity*) to implement case management and actual local implementation.

### Research questions

The study addressed the following questions:

- Have partnerships implemented their case management programs differently from one another?
- What role does willingness to implement play in these differences?
- What role does local capacity play in these differences?

### Data collection

In June 2003, a mail survey was administered to the director of each of New York's 53 partnerships. Directors, rather than case managers, were selected as survey respondents because the directors were responsible for developing new case management programs for local breast screening programs. Thirty-nine (74%) directors responded to the survey. The survey comprised two components: 1) a program philosophy scale adapted by the author for cancer screening case management programs and 2) a set of open-ended questions designed to measure local willingness and local capacity.

**Table:** 

**Box. Subscales Used for Program Philosophy Scale, Survey of Local Breast Cancer Screening Programs, New York, 2003**
Innovation Involvement in job Outreach orientation Team model vs individual case manager model Housing assistance Interest in serving marginalized clients Family orientation Linking to entitlements Emergency access Referral advocacy Interagency orientation Client empowerment philosophy Longitudinality of services
Scale previously was used to assess mental health case management programs (19) and was adapted with permission for this study.

The program philosophy scale was previously used to assess the operating philosophy of mental health case management programs (19) and was adapted with permission for this study. The original scale included the subscales program clarity, cohesion of work group, and supervisory support; these components were eliminated from this study because the unit of analysis for this study was the partnership rather than the individual case manager. Thirteen subscales were used for the program philosophy scale (Box). Each subscale consisted of four statements; two were worded positively, and two were worded negatively. Respondents were asked to indicate whether they strongly disagree, disagree, are neutral, agree, or strongly agree to each of the 56 statements in the survey (Appendix A).

Local willingness to implement case management was assessed with open-ended questions on partnership goals for case management, perceived client needs, tasks to meet client needs, and partnership preferences for case management. Capacity was assessed with questions on perceived barriers to implementation, staffing level, staffing disciplines, case management caseloads, perceived autonomy in designing the case management program, and awareness of recognized case management standards (Appendix B).

### Data analysis

Factor analysis was conducted on subscales of the program philosophy scale to identify underlying common factors that would represent differences in implementation of case management. The consistency within each subscale was assessed using Cronbach's α; subscales lower than 0.60 (interagency orientation and referral advocacy) were eliminated from further analyses. Another subscale (longitudinality of services) had a low communality and also was dropped.

The open-ended questions on willingness and capacity to implement were categorized into themes, coded as dichotomous variables (present or absent), and correlated with the common factors identified in factor analysis. Relationships between some variables that might have been related may not have been detected because of the small number of respondents. *P* values less than .10 were considered significant. SAS-PC software (SAS Institute Inc, Cary, North Carolina) was used in the quantitative analyses.

The institutional review boards of the New York State Department of Health and the University at Albany, State University of New York, approved this study.

## Results

### Partnership implementation

Of the 13 subscales of the program philosophy scale, eight were included in the factor analysis: housing assistance, emergency access, links to entitlements, family orientation, outreach orientation, involvement in job, interest in serving marginalized clients, and innovation. Two common factors — task focus and self-identity focus — were identified. Because these two common factors were somewhat correlated, an oblique rotation was used to aid in data interpretation. Table 1 shows the relationship between the program philosophy subscales and the two common factors.

The first factor — task focus — was so named because it grouped subscales pertaining to the specific tasks of a comprehensive case management program: housing assistance, emergency access, linking to entitlements, family orientation, and outreach orientation. The second factor — self-identity focus — was so named because it grouped subscales pertaining to the partnership's self-perception: involvement in job, interest in serving marginalized clients, and innovation orientation. Self-identity focus reflected the idea of emotional investment in fulfilling a responsibility with creativity and a sense of service to underserved women. One partnership indicated it was "honored" by the women served, who were "poor and have a multitude of problems, yet they are hopeful, strong." Task focus, therefore, reflects *what* partnerships do, whereas self-identity focus reflects self-awareness of *how* the partnership does what it does.

The identification of task focus and self-identity focus as common factors showed that partnerships differed in their approaches to case management. One-third of the partnerships surveyed scored high on both task focus and self-identity focus. Another third scored low on both dimensions. Two-thirds of the partnerships scoring high on self-identity focus also scored high on task focus. The remaining one-third of partnerships were almost evenly divided between high self-identity focus and low task focus and low self-identity focus and high task focus.

### Partnership willingness and capacity to implement

Willingness and capacity to implement were examined as explanations for differences in implementation (Table 2). Task focus was associated with a broader number of goals and tasks, including client support, client empowerment, and health education. Provision of health education correlated with both task focus and self-identity focus. Health education went beyond breast health to include ensuring "that clients have the ability to maximize their use of preventive health services."

Self-identity focus was negatively associated with perceived barriers to implementation, such as time and difficulties with outside agencies or providers. Working systemically to address change on their own was apparent in partnerships that described "networking for pro bono care for both cancer . . . and non-cancer related conditions," undertaking "legislative advocacy for funding," or forming "a networking and continuing education group for professionals." In contrast, task focus was associated with a preference for additional resources, with lack of funding identified as a barrier to implementation.

Having a nurse as the case manager and awareness of established standards of case management both were related to task and self-identity focus. Having a health educator as the case manager was negatively associated with task focus, although health education as a task was strongly related to task focus.

Among nearly all of the partnerships that identified tracking and documentation as a program task, few noted any other tasks or program goals. Partnerships limited to tracking and documentation scored low on both task focus and self-identity focus and were more likely to frame the task as "follow-up with noncompliant clients" or "maintain tracking database." In contrast, self-identity focus was negatively associated (but not significantly so) with identification of tracking and documentation as a program task.

## Discussion

Because case management in partnerships was introduced into an existing program without additional funding to support additional staff, the process and outcome of implementation probably differed from those in a new program. This study identified differences in how partnerships viewed and implemented case management, even though each partnership received consistent messages about case management from NBCCEDP and NYSDOH.

An evolution of case management styles can be inferred from these findings. Partnerships with low scores on both task focus and self-identity focus (one-third of those surveyed) limited themselves to the earlier model of tracking and documentation alone. More than half of partnerships surveyed wished to structure their case management program differently but reported being constrained from doing so, typically by lack of time or funding. In general, partnerships appointed staff members previously assigned to track and follow clients to the new position of case manager.

Task-focus partnerships reported a greater range of case management tasks but also reported the need for additional resources, particularly funding. Nurse case managers or case managers aware of established standards for case management more frequently staffed task-focus and self-identity partnerships. Standards for case management include tasks at all systems levels. Professional nurses may be more familiar with case management standards or may be more accustomed to intervening at multiple systems levels.

To provide case management services beyond tracking, case managers must interact with other agencies. Self-identity partnerships more frequently took on systems-level tasks, describing smooth interactions with other agencies to refer women in need of diagnostic care and treatment and access to "quality of life improvement services such as food stamps," while other partnerships described difficulty "navigating the system, dealing with multiple services and providers." Experience in interacting with other agencies may explain why self-identity partnerships were less likely than task-focus partnerships to report problems in interacting with other agencies.

Despite financial resources comparable with those of other partnerships, self-identity partnerships looked for ways to serve within the constraints under which they worked. Such ways frequently involved acting strategically at multiple systems levels. These partnerships reported satisfaction from their accomplishments, rather than a sense of burden. They are likely to be willing to make changes as they arise or create the capacity themselves, as did one partnership that raised funds within its community and formed a group of local professionals interested in breast health.

This study was conducted at one point in time with a small number of respondents. Consequently, the author cannot claim that knowledge of case management standards led to partnerships acquiring a self-identity focus or that these self-identity partnerships sought information on standards because they already felt involved or perceived themselves as innovators in their jobs. The findings of this study could be strengthened by examining the case management styles of the partnerships and by gaining the perspectives of the directors and case managers since the first data were collected.

Some partnerships cannot be described as having one implementation style, particularly partnerships scoring high on self-identity focus but low on task focus. Some partnership directors may not have provided sufficient detail in answering open-ended questions to completely depict their case management program within this study, or case managers possibly played a role in adapting policy. In-person visits and interviews with case managers and directors would have allowed probing to obtain more program detail.

Helping partnerships improve their understanding of case management standards appears to be one way to build stronger programs; another way is encouraging partnerships to employ or work more closely with nurses. Programs addressing both systems needs and client needs would result.

This study purposely did not examine partnership differences in breast cancer outcomes for several reasons. As noted previously, NYSDOH believed its clients were well-served by the tracking and follow-up services already in place to ensure timeliness of testing, diagnosis, and treatment. The amendment to legislation enabling case management also had the potential to permit states and local programs to more comprehensively approach caring for enrolled women. If states or local programs or both reframe the initial "problem" as not solely breast cancer mortality but also as a lack of continuity of care and the social context of illness as contributors to breast cancer mortality, other health outcomes can be improved along with breast health. Self-identity partnerships that view themselves as involved innovators caring for marginalized clients are better positioned to take such a policy leap.

## Figures and Tables

**Table 1:** Relationship of Program Philosophy Subscales to Task Focus and Self-Identity Focus, Study on Case Management Among New York's Healthy Women Partnerships^a^, 2003

**Program Philosophy Subscale**	**Standardized Regression Coefficients**	

	**Task Focus**	**Self-Identity Focus**
Housing assistance	0.83	−0.13
Emergency access	0.76	−0.01
Linking to entitlements	0.72	0.07
Family orientation	0.65	0.12
Outreach orientation	0.50	0.18
Involvement in job	−0.04	0.83
Interest in serving marginalized clients	0.03	0.68
Innovation	0.08	0.64

a The Healthy Women Partnerships consist of publicly funded breast cancer screening programs organized locally by 53 partnerships made up of more than 2300 partner agencies that provide more than 40,000 mammograms to underserved women annually.

**Table 2:** Univariate Correlations Between Local Willingness and Capacity and Task Focus and Self-Identity Focus, Study on Case Management Among New York's Healthy Women Partnerships^a^, 2003

Model Variable	R^2^ (*P* value)	

	Task Focus	Self-Identity Focus
**Local Willingness to Implement**		
**Partnership goals**		
Client empowerment	0.272 (.10)	0.225 (.18)
Health care provision	0.149 (.38)	−0.205 (.22)
Diagnostic testing	−0.063 (.71)	−0.041 (.81)
**Tasks and needs**		
Client support	0.340 (.04)	0.241 (.15)
Health education	0.334 (.05)	0.348 (.04)
Appointment making	−0.194 (.25)	−0.208 (.22)
Assessing and planning	0.201 (.24)	−0.015 (.93)
Tracking and documentation	−0.202 (.24)	−0.272 (.10)
Referrals	0.087 (.61)	0.23 (.17)
Transportation	−0.099 (.56)	−0.063 (.71)
Assistance with language barriers	−0.050 (.77)	−0.029 (.87)
Financial assistance	−0.003 (.99)	0.124 (.47)
**Preferences for Restructuring Case Management Program**		
Would have additional resources, such as time or staffing	0.293 (.08)	−0.136 (.42)
Would add additional client services	−0.084 (.62)	−0.153 (.37)
Would add services for case managers	−0.123 (.47)	−0.218 (.19)
**Local Capacity to Implement**		
**Perceived barriers to implementation**		
Time	−0.160 (.34)	−0.374 (.02)
Difficulties with outside agencies or providers	0.129 (.45)	−0.320 (.05)
Funding	0.101 (.055)	−0.089 (.60)
Client factors	0.842 (.62)	0.202 (.23)
Staffing	−0.072 (.67)	−0.051 (.77)
Case load	0.068 (.70)	0.062 (.72)
**Staffing discipline of case manager**		
Nurse	0.304 (.07)	0.412 (.01)
Health educator	−0.299 (.07)	−0.002 (.99)
Human services	−0.233 (.16)	−0.245 (.14)
Multidisciplines	0.073 (.67)	−0.189 (.26)
**Awareness of standards of case management**	0.375 (.02)	0.303 (.07)
**Perceived autonomy in implementing case management**	−0.192 (.26)	0.128 (.45)

a The Healthy Women Partnerships consist of publicly funded breast cancer screening programs organized locally by 53 partnerships made up of more than 2300 partner agencies that provide more than 40,000 mammograms to underserved women annually.

## Appendices

### Appendix A. Community Program Philosophy Scale for Cancer Screening Case Management Programs (Adapted from Jerrell and Hargreaves, 1991), New York State Healthy Women Partnerships

Directions: These questions ask about the work style and philosophy practiced by staff in your case management program. Styles often vary with the type of clients served, the personal preferences of both clients and staff, and program traditions — making each program unique. Levels of limitations in funding may also affect style.

*Your program* means the case management component of your Partnership, not all the members of the county Healthy Women Partnership. The scale is intended for understanding introduction of case management into screening programs for medically underserved women.

**THERE IS NO BEST OR CORRECT ANSWER**. This scale is used to provide a general picture of case management within your Partnership. Your individual responses are confidential, but the range of scores for Partnerships across the state will be reported back to you and to staff at NYSDOH. Thank you for your involvement in this project.

Please read each statement and circle whether you strongly disagree; disagree; are neutral; agree; strongly agree. Some items may not seem to apply to your case management program. If the item is not at all true for your program, circle "Strongly Disagree." If you cannot decide whether an item is true or not for your program, circle "Neutral." Please complete every item.

PARTNERSHIP NAME: _______________________________________ Date ____________

**Table:**

**Scale Item**	**Strongly disagree**	**Disagree**	**Am neutral**	**Agree**	**Strongly agree**
01 New and different intervention ideas are being tried out here.	SD	D	N	A	SA
02 Case managers find the work here interesting and challenging.	SD	D	N	A	SA
03 Case managers spend more than half their case management time working with clients out of the office.	SD	D	N	A	SA
04 We use sole case manager assignments rather than a team approach.	SD	D	N	A	SA
05 Helping arrange housing for clients in case management is rarely done here.	SD	D	N	A	SA
06 Case managers find it rewarding and challenging to work with medically underserved clients.	SD	D	N	A	SA
07 We provide little, if any, information or counseling for clients' families.	SD	D	N	A	SA
08 Case managers often help clients obtain income entitlements.	SD	D	N	A	SA
09 The case management program has on-call coverage outside normal work days.	SD	D	N	A	SA
10 When making referrals, a case manager accompanies the case management client on her first contact.	SD	D	N	A	SA
11 Case managers usually work with clients without involving staff from other agencies.	SD	D	N	A	SA
12 We give first priority to being the client's advocate, someone on her side.	SD	D	N	A	SA
13 Case managers see providing good health education messages as the most important case management task.	SD	D	N	A	SA
14 The program emphasizes maintaining long-term regular contact with most case management clients.	SD	D	N	A	SA
15 New ideas about methods of case management are not viewed with enthusiasm here.	SD	D	N	A	SA
16 Case managers seem to be quite involved in their work here.	SD	D	N	A	SA
17 When clients miss appointments we make little effort to keep them involved.	SD	D	N	A	SA
18 Several case managers are assigned to work as a case management team with each client.	SD	D	N	A	SA
19 Housing is seen as the client's individual responsibility and not part of our services.	SD	D	N	A	SA
20 Case managers feel effective in addressing the multiple needs of clients in this program.	SD	D	N	A	SA
21 We teach family members about cancer diagnosis and treatment and what family can do to help.	SD	D	N	A	SA
22 Clients are rarely helped to apply for welfare support.	SD	D	N	A	SA
23 We work closely with hospital staff when one of our clients is treated there.	SD	D	N	A	SA
24 When making referrals, case managers usually allow case management clients to follow through on their own.	SD	D	N	A	SA
25 Case managers spend time ensuring that clients do not get caught in interagency conflicts.	SD	D	N	A	SA
26 Case managers do not support client empowerment or advocacy viewpoints very strongly.	SD	D	N	A	SA
27 Case managers see individual counseling as the most important aspect of working with clients.	SD	D	N	A	SA
28 We help case management clients through a crisis or a transition without continuing to see them indefinitely.	SD	D	N	A	SA
29 The same case management methods have been used here for a long time.	SD	D	N	A	SA
30 The work atmosphere around here is impersonal.	SD	D	N	A	SA
31 We do most of our case management with clients in our office rather than in the field.	SD	D	N	A	SA
32 Our case management team assignments allow staff to be flexibly available to clients in an emergency situation.	SD	D	N	A	SA
33 Case managers will intervene when a client has a housing problem.	SD	D	N	A	SA
34 Case managers prefer to focus most of their work on clients who comply with medical recommendations.	SD	D	N	A	SA
35 Case managers make major recommendations without consulting the family.	SD	D	N	A	SA
36 Our program may take clients to apply for Medicaid.	SD	D	N	A	SA
37 The clients in our case management program do not have a need for us on an emergency basis.	SD	D	N	A	SA
38 Transporting case management clients to needed services is an appropriate case manager activity.	SD	D	N	A	SA
39 Coordinating a multi-agency service plan is rarely done for case management clients here.	SD	D	N	A	SA
40 We systematically seek clients' views about the program.	SD	D	N	A	SA
41 Being educated about the importance of following medical recommendations is our clients' most important need.	SD	D	N	A	SA
42 It is common here for the same case manager or case management team to see clients over many months.	SD	D	N	A	SA
43 There is a fresh, novel atmosphere about this program.	SD	D	N	A	SA
44 Case managers seem to be just putting in time in this program.	SD	D	N	A	SA
45 Working outside of the office is part of our attempt to connect with clients.	SD	D	N	A	SA
46 Clients usually get to know only one case manager really well.	SD	D	N	A	SA
47 Case managers work to insure stable housing for each client.	SD	D	N	A	SA
48 Case managers prefer to work mostly with clients who are willing and able to take care of themselves.	SD	D	N	A	SA
49 In this case management program, families are treated as allies in choosing and delivering services to clients.	SD	D	N	A	SA
50 Case managers rarely help connect clients with non-cancer health care.	SD	D	N	A	SA
51 We advise case management clients and their families to go to the emergency room for crises outside normal work hours.	SD	D	N	A	SA
52 Helping case management clients with the application process in other agencies is rarely done here.	SD	D	N	A	SA
53 Case managers give high priority to resolving interagency disagreements about a client's needs.	SD	D	N	A	SA
54 Case managers make major case management decisions without consulting the client.	SD	D	N	A	SA
55 Clients need case management because they face problems in life besides cancer screening test results.	SD	D	N	A	SA
56 Most clients here receive case management over a period of a few weeks.	SD	D	N	A	SA

### Appendix B. Additional Questions for Cancer Screening Case Management Programs, New York State Healthy Women Partnerships

As a Healthy Women Partnership Director, what is your professional training? (Check all that apply):

____ Nurse (RN)

____ Nurse Practitioner/Advanced Practice Nurse (master's level)

____ Social Worker (BSW)

____ Social Worker (MSW)

____ Counselor (BA)

____ Psychologist (PhD)

____ Physician (MD)

____ Other (please specify: ___________________________________________)

Please list the number of full-time and part-time case managers within your Partnership by their primary professional discipline in the table below:

**Table:**

**Staffing Disciplines**	**Number of Full-time Case Managers by Discipline**	**Number of Part-time Case Managers by Discipline**
Nurse (RN)		
Nurse Practitioner/Advanced Practice Nurse (masters level)		
Social Worker (BSW)		
Social Worker (MSW)		
Counselor (BA)		
Psychologist (PhD)		
Physician (MD)		
Other (please specify):		

SCREENING CASELOAD PER YEAR: ______________________ women

CASE MANAGEMENT CASELOAD PER YEAR: ______________________ women

What are your objectives for your Partnership's case management program?

_______________________________________________________

_______________________________________________________

_______________________________________________________

What are the major activities/tasks you employ in your Partnership's case management program?

_______________________________________________________

_______________________________________________________

_______________________________________________________

What kinds of client needs are typically addressed in your case management program?

_______________________________________________________

_______________________________________________________

_______________________________________________________

How far along would you say you are in implementing your case management program?

_____ Fully implemented

_____ In the process of implementing

_____ Planning implementation

Have you experienced any barriers to implementing your case management program? If so, what?

_______________________________________________________

_______________________________________________________

_______________________________________________________

If you could, would you structure your case management program differently? How? What factors prevent you from doing so?

_______________________________________________________

_______________________________________________________

_______________________________________________________

_______________________________________________________

_______________________________________________________

_______________________________________________________

Are you familiar with standards of case management practice such as those prepared by National Association of Social Workers or the Case Management Society of America?

_____ Yes

_____ No

_____ Unsure

To what extent do case managers in your Partnership utilize these standards?

_______________________________________________________

Why or why not?

_______________________________________________________

Other comments:

_______________________________________________________

_______________________________________________________

_______________________________________________________

_______________________________________________________
